# Evidence for a Common Genetic Origin of Classic and Milder Adult-Onset Forms of Isolated Hypogonadotropic Hypogonadism

**DOI:** 10.3390/jcm8010126

**Published:** 2019-01-21

**Authors:** Biagio Cangiano, Paolo Duminuco, Valeria Vezzoli, Fabiana Guizzardi, Iacopo Chiodini, Giovanni Corona, Mario Maggi, Luca Persani, Marco Bonomi

**Affiliations:** 1 Department of Clinical Sciences and Community Health, University of Milan, 20100 Milan, Italy; biagio.cangiano@live.com (B.C.); iacopo.chiodini@unimi.it (I.C.); luca.persani@unimi.it (L.P.); 2 IRCCS Istituto Auxologico Italiano, Division of Endocrine and Metabolic Diseases & Lab. of Endocrine and Metabolic Research, 20149 Milan, Italy; p.duminuco@auxologico.it (P.D.); valeria.vezzoli@gmail.com (V.V.); f.guizzardi@auxologico.it (F.G.); 3 Endocrinology Unit, Medical Department, Azienda USL, Maggiore-Bellaria Hospital, 40133 Bologna, Italy; jocorona@libero.it; 4 Department of Biomedical, Experimental and Clinical Sciences “Mario Serio”, University of Florence, 50139 Florence, Italy; m.maggi@unifi.it

**Keywords:** GnRH, Kallmann’s Syndrome, Late onset hypogonadism, obesity, IHH, testosterone cutoff, BMI, oligogenicity

## Abstract

Multiple metabolic and inflammatory mechanisms are considered the determinants of acquired functional isolated hypogonadotropic hypogonadism (IHH) in males, whereas classic IHH is a rare congenital condition with a strong genetic background. Since we recently uncovered a frequent familiarity for classic IHH among patients with mild adult-onset hypogonadism (AO-IHH), here we performed a genetic characterization by next generation sequencing of 160 males with classic or “functional” forms. The prevalence of rare variants in 28 candidate genes was significantly higher than in controls in all IHH patients, independently of the age of IHH onset, degree of hypogonadism or presence of obesity. In fact, it did not differ among patients with classic or milder forms of IHH, however particular genes appear to be more specifically associated with one or the other category of IHH. ROC curves showed that Total Testosterone <6.05 nmol/L and an age of onset <41 years are sensitive cutoffs to identify patients with significantly higher chances of harboring rare IHH gene variants. In conclusion, rare IHH genes variants can frequently predispose to AO-IHH with acquired mild hormonal deficiencies. The identification of a genetic predisposition can improve the familial and individual management of AO-IHH and explain the heritability of congenital IHH.

## 1. Introduction

Isolated hypogonadotropic hypogonadism (IHH) is currently known as a rare congenital disorder which has a male predominance of 3–5 to 1 [1,2,3,4]. It consists of an isolated gonadotropin-releasing hormone (GnRH) secretion, regulation, or action defect in the presence of an otherwise normal hypothalamic–pituitary region, which leads to a delayed or absent puberty and infertility [2,5]. The same disease can manifest either with a normal sense of smell (normosmic IHH, nIHH), or hypo-anosmia (Kallmann syndrome, KS), having these last group of patients usually a more severe phenotype [1,2,3,6]. Due to the reduced fertility, this disease was thought to be essentially sporadic, but several familial cases have been described and the existence of an intra- and inter-familial variable penetrance and expressivity, including the fertility trait, has become evident [5,7]. Indeed, even if the causes of IHH remain often unsolved [5], several evidence outlined the importance of a genetic background. Presently, IHH is associated to predisposing allelic variants in genes required for development and migration of GnRH cells, for their activation, for the secretion of the GnRH itself, or for the mediation of its effect. Genetic analysis can explain no more than 45%–50% of the cases [1] and the number of involved loci is constantly increasing. Moreover, it is recognized that a portion of congenital IHH can be explained by an oligogenic origin [8], which can potentially account for the variable penetrance and expressivity. 

IHH is classically diagnosed at birth or pubertal delay (Pre-pubertal onset IHH, PPO-IHH) due to defective masculinization and confirmed by low total testosterone levels (TTe < 3.5 nmol/L), as reported in the initial description of the syndrome [9,10,11]. However, adult onset IHH with similar hypotestosteronemia (TTe < 4–4.5 nmol/L) and variable fertility have also been reported as post-pubertal IHH cases (Adult onset Hypogonadotropic Hypogonadism, AO-IHH), thus giving a potential justification for the existence of IHH families [12,13]. AO-IHH has been associated with minor pubertal delays, thus suggesting an underlying preexisting mild impairment of hypothalamic–pituitary–gonadal (HPG) axis [12] and a multifactorial etiology of the disease.

We recently reported AO-IHH patients with familiarity for congenital IHH and related extra-reproductive clinical manifestations [3]. Among AO-IHH patients, including patients with milder forms of hypogonadism (testosterone < 8 nmol/L), we also found a higher prevalence of obesity compared to PPO-IHH patients or general population. Presently, obesity is regarded as a trigger of functional or “false” hypogonadism, however it is unclear if an increase in BMI could have a role in favoring the hormonal defect in predisposed individuals. 

In this work, we investigated the genetic predisposition in a large number of IHH individuals using a comprehensive NGS panel and analyzed the distribution and enrichments of genetic variations upon the age of IHH onset, the degree of hypotestosteronemia and the presence of obesity. 

## 2. Patients and Methods

### 2.1. Editorial Policies and Ethical Considerations

The study, accomplishing the Declaration of Helsinki, was approved by the Ethic Committee of the coordinating institution (GR-2008-1137632), and all patients or their tutors gave a written informed consent.

### 2.2. Study Population

The entire cohort consists of 160 unrelated males recruited by the NICe group since 2008. Anonymous patient data, referred to the time of diagnosis, before any therapy, were collected either prospectively or retrospectively and a clinical database was created. All subjects were affected with IHH (age range at diagnosis: 13–70 years), including patients with normal (*n* = 101) or olfactory defects (hypo- or anosmia, *n* = 59) as demonstrated using Brief Smell Identification Test (B-SIT), MRI, or both. IHH was defined as: (1) manifestations of hypogonadism associated with low testosterone and inappropriately low/normal gonadotropins; (2) absence of any known acquired IHH cause (i.e., expansive hypothalamic/pituitary lesions, hemochromatosis, etc.), or multiple pituitary hormone defects (MPHD). In order to omit the functional hypothalamic defects, exclusion criteria were: (1) severe weight loss (body mass index < 18.5 kg/m^2^) [14]; (2) intensive exercise (>5 hours/week); (3) chronic illness and psychiatric disorders. 

Patients were sub-divided according to age of onset and degree of hypotestosteronemia as follows. The patients who did not enter or complete spontaneous pubertal development were classified as PPO-IHH (age range at diagnosis: 14–37 years). Whereas, the adult patients who had completed their pubertal development (bitesticular volume > 24 mL) but presented with (a) loss of libido, (b) erectile dysfunction, (c) loss of spontaneous nocturnal erections, (d) repeatedly low testosterone with low/normal gonadotropins were classified as AO-IHH (age range at diagnosis: 25–70 years). Patients diagnosed with IHH during adolescence were reexamined after therapy withdrawal between 17 and 20 years, in order to exclude a constitutional delay of puberty. We studied 110 patients with TTe below the classic value of 3.5 nmol/L (here addressed as severe IHH, sIHH) and 50 patients with TTe >3.5 but <8 nmol/L or <11 nmol/L but with calculated free testosterone (cFT) <220 pmol/L (here referred as mild IHH, mIHH). Among the entire cohort 109 patients had a PPO-IHH (100 patients with a sIHH and 9 with a mIHH) and the remaining 51 an AO-IHH (10 patients with a sIHH and 41 with a mIHH). 

Finally, the 26% of the patients studied were obese as defined by a BMI value >30 [14] whereas the remaining 74% had a BMI ranging 19–29.9 kg/m^2^. Clinical characteristics of our cohort are reported in Table 1. All basal blood samplings were performed before 9:00 a.m., after an adequate fasting period, and the low hormonal levels were confirmed on at least two separate, consecutive, determinations.

### 2.3. Assays

Because we recruited patients with a diagnosis obtained up to 25 years ago, different methods had been used. In the majority of the cases, serum LH, FSH, and Testosterone concentrations were measured by electrochemiluminescence immunoassay “ECLIA” from Roche Diagnostic (Roche Diagnostics GmbH, Germany). LH and FSH assays had a lower limit of detection of 0.1 IU/L and a functional sensitivity of 0.2 IU/L. Elecsys^®^ Testosterone II test (Calibrator reference: 05200067 190, Rotkreuz, Swiss) had a lower limit of detection of 0.087 nmol/L and a functional sensitivity of 0.4 nmol/L. The inter- or intra-assay coefficients of variation were <5% in all assays since 2008. Steroid method was standardized via isotope dilution-gas chromatography/mass spectrometry. SHBG levels were measured using a solid-phase, chemiluminescent immunometric assay on Immulite 2000 (Medical Systems Corp., Genoa, Italy), with a lower limit of detection of 0.2 nmol/L and the intra- and interassay coefficients of variation of <5% and <6%, respectively.

### 2.4. Genetic Analyses by Targeted Next Generation Sequencing (NGS)

Each patient underwent a genetic investigation, using a targeted NGS technique, to search for rare allelic variants. We extracted the genomic DNA of each patient from peripheral blood lymphocytes using Gene Catcher gDNA 96 × 10 mL Automated Blood kit (Invitrogen, Life TechnologiesTM, Carlsbad, CA, USA). The IHH gene panel was designed using Illumina Design Studio (San Diego, CA, USA) and included the following IHH candidate genes: *ANOS1(KAL1), FGFR1, PROKR2, PROK2, GNRHR, GNRH1, GNRH2, KISS1, KISS1R, TAC3, TACR3, HS6ST1, FGF8, CHD7, DUSP6, FEZF1, FGF17, FLTR3, IL17, SEMA3A, SEMA3E, SEMA7A, SOX2, SOX10, SPRY4, WDR11, HESX1, NELF*. The 28 IHH genes consistently represented in all sequence capture panels were assessed for the purposes of this study. Libraries were prepared using Illumina Nextera Rapid Capture Custom Enrichment kits according to the manufacturer’s protocols. All regions not correctly sequenced were recovered with NexteraVR DNA Library Preparation kit (Illumina, San Diego, CA, USA). For subsequent analyses, we included as “rare variants” [15] all known pathogenic, or rare non-synonymous or splicing-site variants (Minor Allele Frequency, MAF ≤ 0.01) and novel non-synonymous or splicing-site variants. The frequency and the functional annotation of the identified variants were checked in public and licensed databases (Ensembl, UCSC Genome browser, 1000 Genome project, ExAC Browser, NCBI, HGMD professional), considering the ethnic groups (Europeans). As previously reported [16], we excluded common non-synonymous variants with Minor Allele Frequency (MAF) >0.01, synonymous, intronic, and 5′ or 3′ UTR variants. Each variant found was confirmed by Sanger direct sequencing using BigDyeVR Terminator v.3.1 Cycle Sequencing Kit (Life Technologies, Carlsbad, CA, USA) on a 3100 DNA Analyzer from Applied Biosystems (Foster City, CA, USA). We performed the same NGS analysis in 79 controls from the general population admitted for blood donation (52.9% males; age range: 18–70 years, mean 33.9; BMI range: 18–30 kg/m^2^, mean 23.3).

### 2.5. Statistical Methods

Statistical analysis was performed by SPSS, version 21.0, statistical package (SPSS Inc., Chicago, IL, USA). The data obtained were analyzed with Fisher’s exact test to compare the prevalence of mutations in the causal genes of IHH affecting PPO-IHH with those affecting AO-IHH and controls. Next step was the comparison of the prevalence of rare variants (including all single variants, either those presenting as monogenic or those associated with other variants in an oligogenic pattern) between patients with severe hormone deficiencies, patients with mild/moderate hormone deficiency and in controls; finally, we also compared the prevalence of these mutations in obese HH patients with such prevalence in non-obese hypogonadotropic subjects and controls. We also performed a receiver operating characteristic (ROC) curve analysis in order to assess the cutoff values of total testosterone levels, age and BMI with a sensitivity higher than 90% for predicting the presence of rare variants in the causal genes of IHH; we than compared the patients again according to these results. Finally, we evaluated the presence of oligogenicity (intended as the finding of rare variants in more than one gene) among the groups using the full panel, and characterized the genotype in each category of patients.

## 3. Results

The overall percentage of patients harboring rare variants, considering the panel of 28 genes was 55% (*n* = 88), whereas it was 32% (*n* = 51) when we restricted the analysis to the 13 firstly discovered (“classic”) IHH genes (*ANOS1, FGFR1, PROKR2, PROK2, GNRHR, GNRH1, GNRH2, KISS1, KISS1R, TAC3, TACR3, HS6ST1, FGF8*). We then performed comparisons between variable groups of patients, divided according to the abovementioned parameters, and controls both considering the full panel of genes and the restricted evaluation.

### 3.1. Rare Allelic Gene Variants Enrichment According to TTe Levels, IHH Onset, Obesity, Olfactory Defects 

**Figure 1:**
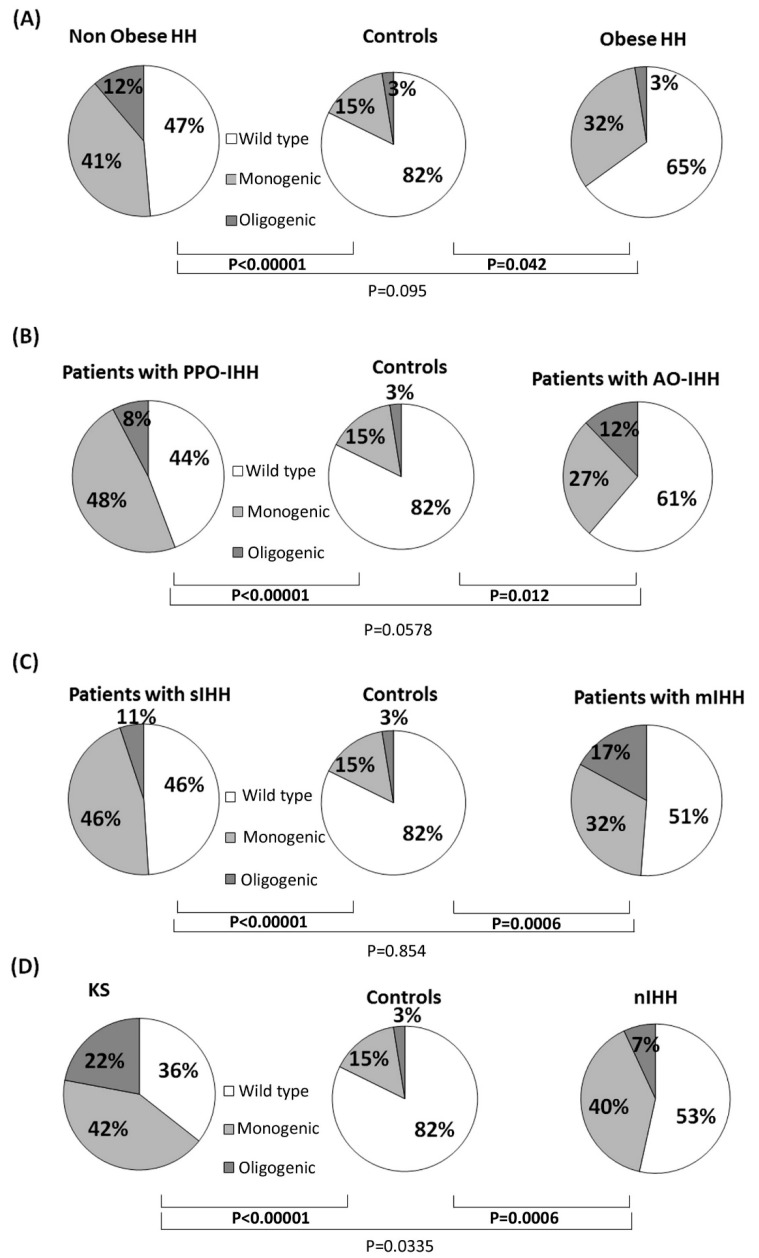
Frequency of rare genetic variants in 28 IHH candidate genes among the different groups of patients and controls. Comparisons of rare allelic variants prevalence according to the presence of obesity (**A**), age of IHH onset (**B**), TTe levels (**C**) and olfactory defects (**D**). “Rare variants” includes all single variants, either those presenting as monogenic or those associated with other variants in an oligogenic pattern.

The prevalence of rare variants (as defined in “statistical methods”) was significantly higher than in controls in both patients with sIHH (<3.5 nmol/L) or mIHH, while no relevant differences were noticed between the two patients’ groups (Figure 1). Comparable results were obtained also with a less strict testosterone cutoff for mIHH (mIHH: TTe < 4.5 nmol/L): mIHH vs. controls *p* = 0.0018; sIHH vs. controls *p* < 0.00001; sIHH vs. mIHH *p* = 0.57. The patients with mIHH appeared more likely to have an oligogenic involvement when compared to controls (*p* = 0.0074), but there was not any significant difference between sIHH and mIHH. 

When the analysis was performed according to the onset of the disease, both the PPO-IHH and AO-IHH patients showed a similar and statistically significant higher prevalence of rare variants than controls (Figure 1). We found no relevant differences in the prevalence of oligogenic defects in the two groups of cases (*p* = 0.38) and controls (*p* = 0.19 with PPO-IHH, *p* = 0.053 with AO-IHH). 

When the analysis was performed according to the BMI, a number of rare gene variants significantly higher than in controls was found in both obese and non-obese patients, while no significant differences were observed comparing the two groups of cases (Figure 1). Oligogenic defects appeared more likely to occur in non-obese hypogonadal patients with a significantly higher prevalence than in controls (*p* = 0.0265).

As a positive control, we performed the same analysis according to the presence of olfactory defects: we observed that both KS patients and normosmic-IHH had an increased prevalence of rare variants in IHH genes compared to healthy subjects (*p* < 0.0001), but KS patients had a significantly higher enrichment than nIHH (*p* = 0.033).

**Figure 2:**
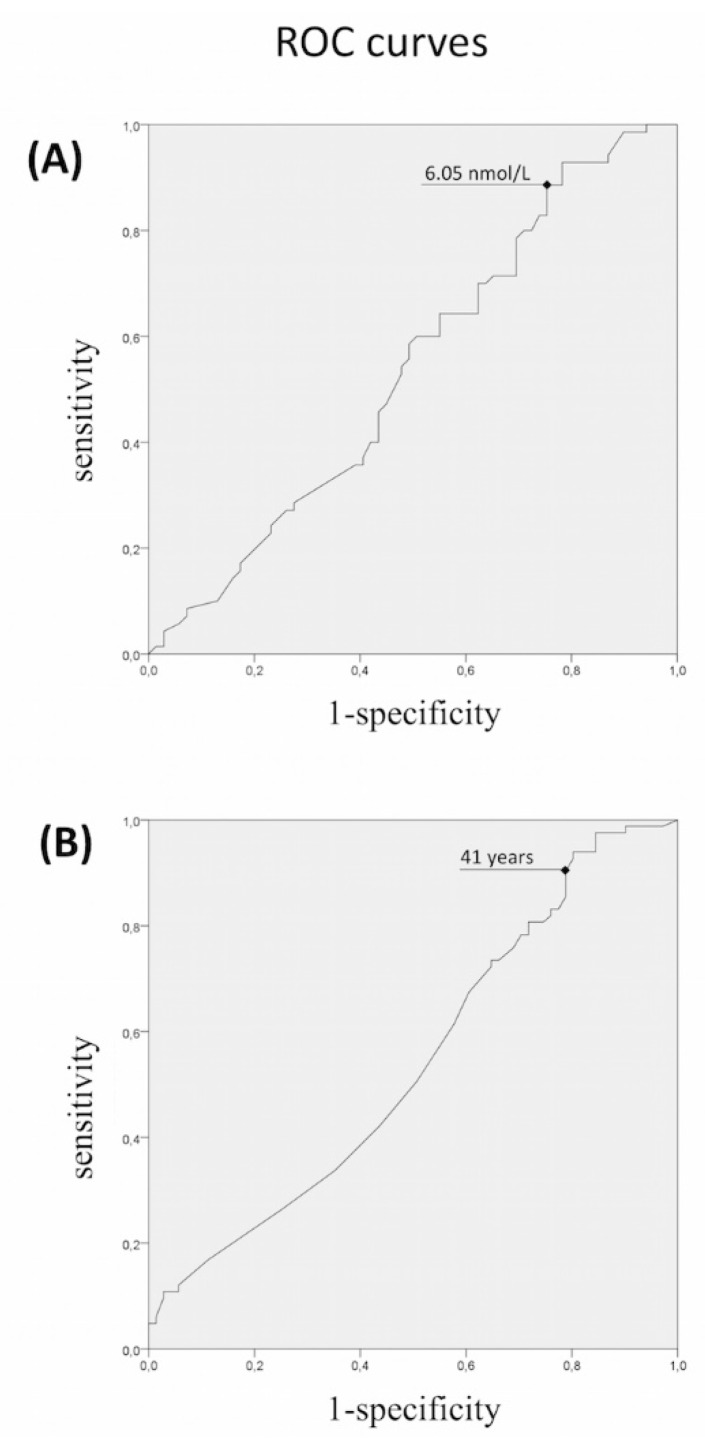
Receiver-operating characteristic (ROC) analysis of (**A**) total testosterone levels, and (**B**) age of onset, as indicators of the presence of rare variants in IHH genes. ROC curves were not significant “per se” but were used to find sensitive cutoffs able to identify patients with significantly higher risk of carrier status.

We finally calculated the receiver-operating characteristic curves to identify cutoffs with a high sensitivity to find patients harboring rare variants (Figure 2). The ROC curves identified a cut-off for TTe values of <6.05 nmol/L and for the estimated age of onset <41 years, with a sensitivity above 90% (specificity 22% and 20%, respectively), for predicting the presence of rare variants.

When the analysis of rare variants was performed according to these thresholds, the patients presenting these characteristics had a significantly higher chance to harbor rare IHH variants when compared with the other patients (*p* = 0.016 and *p* = 0.04 respectively). We could not find a BMI value able to significantly distinguish patients with a higher probability of harboring rare variants. 

Since this TTe threshold is almost 100% higher than the standard criteria for the diagnosis of congenital IHH reported in literature [9,10,11], we decided to perform the same analyses with a restricted panel of 13 “classic IHH genes”. In this case, the ROC curve revealed a TTe cutoff of 3.9 nmol/L as value with a sensitivity ≥91% in finding rare gene variants, similar to the value adopted as classic diagnostic criteria (3.5 nmol/L). 

Thus, we performed the comparisons with Fisher exact test (already carried out using the full 28 gene panel) between patients grouped according to classic TTe thresholds, onset of disease, obesity, and controls, evaluating only the restricted panel.

The NGS analysis of the 13 “classic IHH genes” showed a significant enrichment in rare variants in patients with sIHH compared to both controls (Fisher exact test; *p* < 0.00001) and mIHH (*p* = 0.011), whereas no significant differences were seen between mIHH and controls (*p* = 0.12). Additionally, both PPO-IHH (*p* < 0.00001) and AO-IHH (*p* = 0.043) had a higher prevalence of rare variants compared to controls, but there was a statistically significant difference even between the two groups of cases with a significantly higher prevalence of variants in the 13 “classic IHH genes” in PPO-IHH (*p* = 0.007). Obesity did not reveal again to be a discriminant trait and both obese and non-obese patients with IHH were enriched in rare variants compared to controls (*p* = 0.0051 and *p* < 0.00001, respectively), independently of the age of onset and severity.

### 3.2. Differences in Gene Involvement According to Testosterone Level, IHH Onset, Obesity, Olfactory Defects 

**Figure 3:**
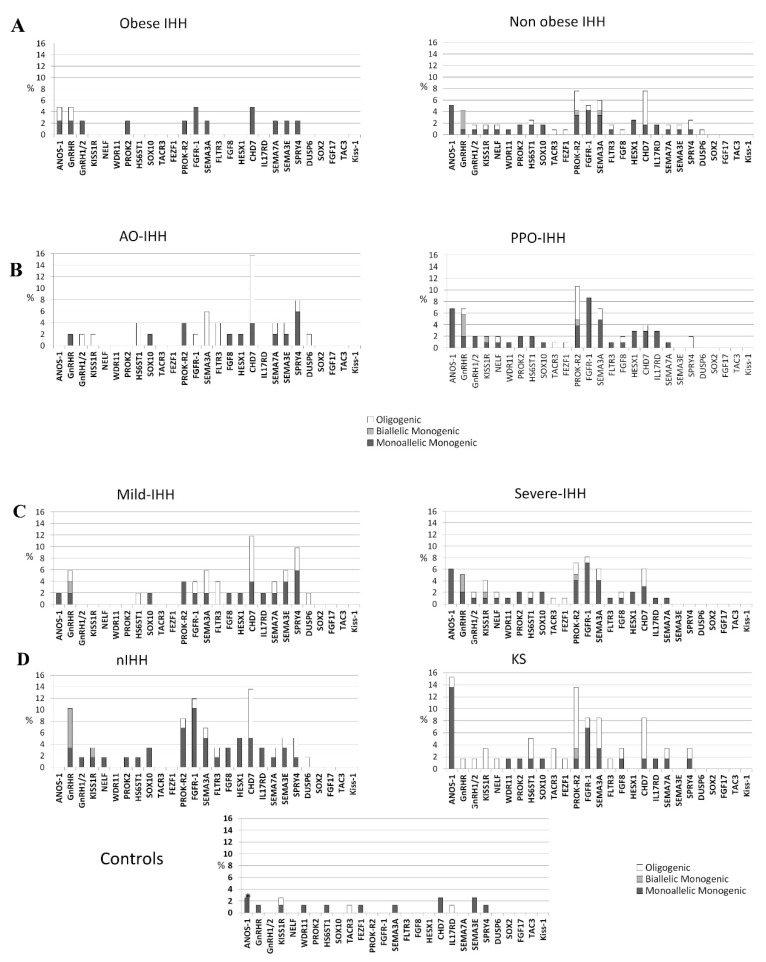
Distribution of rare variants among the 28 candidate genes in the different IHH groups. Showed according to the presence of obesity (**A**), age of IHH onset (**B**), TTe levels (**C**) and olfactory defects (**D**). IHH (isolated hypogonadotropic hypogonadism), AO (adult onset), PPO (prepubertal onset), mIHH (mild IHH), sIHH (severe IHH), KS (Kallmann’s Syndrome), nIHH (normoosmic IHH). Rare variants of *ANOS1* were found only in two female controls; no male subject had a rare variant in *ANOS1* among controls.

Genetic profiling of IHH groups showed large overlaps between patients presenting mIHH and those having AO-IHH. These two categories include several cases harboring *SPRY4* and *SEMA3E* rare variants that are instead absent among sIHH and PPO-IHH. In contrast, rare allelic variants of *ANOS1* and *FGFR1* or biallelic variants in *GnRHR* and *PROKR2* are more represented in sIHH and PPO-IHH groups (Figure 3). *CHD7, SEMA3A, FLTR3*, and *PROKR2* heterozygous mutations seem to be equally distributed among the four different groups (PPO-IHH, AO-IHH, sIHH, mIHH). As expected on the basis of previous literature, only patients with olfactory defects were enriched with variants in *ANOS1*, whereas heterozygous *GnRH* or *GnRHR* gene variants were found only in an oligogenic context. Also, this analysis revealed no relevant difference between obese and non-obese patients (Figure 3). Accordingly, we report many patients having anosmia, severe hypotestosteronemia and prepubertal onset hypogonadism associated to rare variants in IHH genes, despite the presence of obesity, thus confirming the poor utility of this parameter to exclude an “organic” disease (Supplementary Table S1).

Confirming our epidemiological data, among patients presenting with mild impairment of testosterone levels or adult onset disease, or both, we found many variants whose pathogenicity has already been assessed with functional evaluations or had already been associated to the classic phenotype [11,17,18,19,20,21,22,23,24,25]. In particular, in three patients presenting with mild, adult-onset disease we found variants (*PROKR2* R85H, FGF8 P26L, and *HS6ST1* R382W) already reported to impair protein activity in several functional characterization or to segregate with the phenotype, or both [26,27,28,29,30,31,32,33]. Lastly, a variant of *GnRHR* (Q106R) found in homozygosity in a patient with mild hormonal defect (see supplementary data), was already reported in literature as pathogenic for classic IHH [18,19,20,21,22,24] (Supplementary Table S1).

Finally, we analyzed all these variants found in classic and non-classic phenotypes of IHH, as well as in our control group, with common prediction software considering as deleterious (disruptive AVs) every variant with four negative prediction out of six tools used (see Supplementary Tables S1–S3) and nonsense or frameshift variants leading to a premature stop codon. This evaluation showed that both classic and non-classic forms of IHH were enriched in disruptive AVs respect to controls (42/68 variants found in classic IHH; 25/48 variants found in non-classic IHH patients; 4/16 in controls).

## 4. Discussion 

The present study, to our knowledge, is the first extensively evaluating the genetic predisposition in IHH subjects sub-grouped depending upon various traits and severity of hypotestosteronemia, thus considering the wide spectrum of the phenotypic manifestation of this neuroendocrine defect [1,3,34]. We evaluated whether the current published criteria for diagnosis of IHH [1,9,10,11] are able to predict a higher prevalence in rare variants in the IHH candidate genes, both considering a large panel of 28 genes and a restricted panel of 13 “classic IHH genes” excluding the pathway of semaphorins and the last discovered genes of the *FGF8-FGFR1* synexpression group. We checked the possible existence of cutoffs of total testosterone, age of onset, and BMI values having a higher sensitivity in predicting patients with IHH harboring rare genetic variants. Our findings demonstrate that patients at higher risk of harboring rare variants in the 13 “classic IHH genes” have TTe levels below the published cutoffs of 3.5 or 4 nmol/L [9,10,11,12,13] whereas such cutoff cannot predict the risk of variants detection using the full panel of 28 candidate genes. The evaluation of all the 28 candidate genes revealed that the most specific, highly sensitive TTe cutoff rises up to 6.05 nmol/L. This cutoff threshold offers an “acceptable” specificity and an optimal sensitivity, i.e., the ability to detect rare variants predisposing to IHH in a disease population. As a consequence, if a more stringent TTe cutoff would be used, several families harboring genetic defects determining hypogonadism and subfertility/infertility would remain undiagnosed, thus hampering the possibility of an early diagnosis for the affected individuals among their relatives. Therefore, the variable penetrance of particular genetic defects represents a suitable explanation for the existence of milder phenotypes and the unexpectedly high percentage of inherited vs. de novo genetic variants in IHH patients, despite the associated fertility defect. Moreover, since the subjects in the control group are younger than 48 years of age, we cannot predict whether some of them will develop the IHH phenotype in the future. 

The control group of blood donors analyzed with the same NGS panel could not be matched for age, sex, and weight. However, age and sex (which is mixed in the control group) are variables that cannot not be responsible of a lower enrichment in germinal rare variants. An interfering role of BMI is also unlikely as the disrupting variants in IHH genes here considered should not represent a cause of obesity independent of hypogonadism. 

As far as the age of IHH onset is concerned, our analysis showed that the limit of 41 years represents a sensitive cutoff able to detect patients with a significantly higher risk of rare genetic variants in IHH candidate genes. In contrast, we could not find any BMI value associated with a higher enrichment of rare allelic variants. This suggests that obesity alone would not be sufficient to cause IHH. Consistently, only a portion of male obese subjects develops IHH during their life, thus obesity could be only one of the acquired cofactors involved in the onset of IHH among adult subjects that are naturally prone to develop a central failure of the gonadal axis. These AO-IHH subjects are frequently carrying susceptibility alleles with an impact on GnRH function that alone are not able to manifest as pre-pubertal IHH. If confirmed and expanded, these findings provide a strong support to the idea of a multifactorial origin of IHH in which genetic abnormalities should have a variable impact on the disease. Various combinations of oligogenic defects, each one with a minor functional impact on GnRH function, appear more frequent among patients with milder hypogonadism lacking a major damage of the axis; however, their prevalence is higher in normal-weight patients as if a major genetic damage is required in the absence of a metabolic disorder. Therefore, beside the existence of monogenic forms with congenital onset, genetic variants with a minor pathogenic impact may cumulate and interact with other acquired factors (such as obesity) in determining the phenotype. In this genome–environment interaction, rare gene variants could not be responsible of a dichotomous outcome (disease–not disease) but rather of a frailty, based on the number of loci affected and the degree of genetic damage. With this in mind, the detection of a congenital frailty of the hypothalamic–pituitary–gonadal axis would represent an advantage also in obese patients with a milder phenotype, since this may identify the AO-IHH patients that could benefit of a testosterone replacement together with lifestyle changes. In particular this could lead to a better outcome of their obesity rehabilitation programs. 

Hence, our results underline the existence of a mild form of GnRH deficiency characterized by a genetic origin that is frequently overlapping with that of the severe IHH forms. Indeed, looking at the enrichment of rare IHH alleles we observed a statistically significant difference between patients and controls, independently of the severity/onset of their hormonal defect, while we could not observe any relevant difference between sIHH/mIHH and PPO-IHH/AO-IHH comparisons (Figure 1). Moreover, subjects with AO-IHH and/or mIHH deficits showed a lower prevalence of rare variants in the 13 firstly discovered (classic) IHH genes, in particular the absence of hemizygous variants in *ANOS1*, or biallelic variants in *GnRHR* and *PROKR2*, while they presented a higher prevalence of newly discovered genes such as *SEMA3E* and *SPRY4* (Figure 3). Such differences could be attributed to a variable requirement of these genes for GnRH function and the absence of compensating factors for the complete loss-of-function of particular genes (such as *ANOS1* and *GnRHR*).

Limitations of this study are the lack of functional evaluation of the identified variants and segregation studies in affected families, as we are aware that all rare variants may not be disease-causing per se, but may represent a predisposing condition in a multifactorial origin of the disease. Nevertheless, performing the same NGS panel analysis in all groups of patients and controls from the same ethnicity, the statistically significant enrichment in rare variants found in cases supports the conclusion that these variants may contribute the pathogenesis. This view is confirmed by the analysis in another very large population database (GnomAD) filtering for the European non-Finnish population. This additional analysis confirmed the previous findings since only one variant shifted from rare (MAF < 0.01) to low frequency (MAF 0.01–0.05) thus not changing our overall results.

## 5. Conclusions

In conclusion, severe and mild forms of IHH have a largely overlapping genetic origin. Monogenic forms, such as hemizygous ANOS1 mutations or biallelic *GnRHR* or *PROKR2* variants, can explain the more severe phenotypes whereas various combinations of minor IHH alleles with or without acquired factors, such as obesity, can justify the adult-onset of IHH. The analysis of the genetic results in combination with specific traits indicate to offer an extensive genetic analysis to patients who received a proven diagnosis of IHH with a classical pre-pubertal onset, but also if they presented an adult-onset before 41 years of age and total testosterone circulating levels <6.0 nM, independently if they are obese or present as sporadic cases. 

## Figures and Tables

**Table 1:** Cohort clinical characteristics.

Sub-Groups	*n* (%)	Mean LH U/L (SD)	Mean FSH U/L (SD)	Mean Total Testosterone nmol/L (SD)	Mean Age at Diagnosis Years (SD)	Mean Bitesticular mL at Diagnosis (SD)	Mean BMI at Diagnosis (SD)	Hearing Defects	Renal Agenesia	Familiarity	Synkinesias	Midline Defects
**PPO-IHH**	109 (68%)	0.7 (±0.8)	1.1 (±1)	1.4 (±1.5)	17.4 (±6.4)	5.6 (±3.3)	24.7 (±5.3)	2%	2%	30%	9%	15%
**AO-IHH**	51 (32%)	1.7 (±1.2)	2.8 (±2.1)	5 (±2.3)	37.7 (±13.9)	35.9 (±8.2)	28.9 (±5.8)	0%	0%	2%	2%	0%
**sIHH**	110 (69%)	0.7 (±0.8)	1 (±0.9)	1 (±0.8)	18 (±6.9)	7.7 (±7.9)	24.4 (±4.8)	2%	2%	30%	9%	13%
**mIHH**	50 (31%)	1.7 (±1.2)	2.9 (±2)	5.8 (±1.5)	36.9 (±14.9)	32 (±13.7)	29.7 (±6)	0%	0%	3%	3%	3%
**Obese**	42 (26%)	1.1 (±1.1)	2 (±2.1)	3.6 (±2.5)	29.1 (±14.9)	21.4 (±16)	34 (±3.7)	0%	0%	2%	5%	5%
**non Obese**	118 (73%)	0.95 (±1)	1.5 (±1.4)	2.2 (±2.4)	22.1 (±12.3)	13.1 (±14.2)	23.2 (±3.1)	2%	2%	30%	7%	10%

All the subgroups come from the total cohort of 160 patients, grouped in each evaluation according to either bitesticular volume at diagnosis (AO-IHH if BTV >24 mL), severity of hormonal defect (sIHH if TTe at diagnosis <3.5 mmol/L), or presence of obesity. Gonadotropin normal basal. Values are (IU/L): LH > 1.7; FSH > 1.5. Total Testosterone normal basal values are: 9.9–27.8 nmol/L. PPO-IHH: bitesticular volume at diagnosis <24 mL; AO-IHH: bitesticular volume at diagnosis> 24 mL; sIHH: Total Testosterone <3.5 nmol/L; mIHH Total testosterone > 3.5 and <8 or <11 nmol/L but with calculated free testosterone (cFT) <220 pmol/L.

## Supplementary Materials

The following are available online at http://www.mdpi.com/2077-0383/8/1/126/s1, Table S1: Gene allelic variant list in non-classic IHH patient group, Table S2: Gene allelic variant list in classic IHH patient group, Table S3: Gene allelic variant list in controls.
