# Supplementary material for: Evidence for a Common Genetic Origin of Classic and Milder Adult-Onset Forms of Isolated Hypogonadotropic Hypogonadism
Source: J Clin Med. 2019 Jan 21;8(1):126. doi: 10.3390/jcm8010126 (PMC6352096; doi:10.3390/jcm8010126)
Supplement: Supplementary file 1 [file jcm-08-00126-s001.zip › Supplementary Table S1.docx]

**Table S1.** Gene allelic variant list in non-classic IHH patient group.

| **ID** | **Gene** | **Olfactory defect** | **Testosterone deficit** | **Disease Onset** | **Obesity** | **Variant** | **HGVS** | **SIFT** | **Polyphen2** | **LRT** | **MT** | **MA** | **FATHM** | **SCORE** |
| --- | --- | --- | --- | --- | --- | --- | --- | --- | --- | --- | --- | --- | --- | --- |
| 1 | SPRY4 | nIHH | m | AO | no | R91W | *ENST00000344120.4:c.271C>T* | D | B | N | D | L | T | 2/6 |
| 2 | CHD7 | nIHH | m | AO | no | K686-T687insAK | *new* | . | . | . | . | . | . |  |
|  | SPRY4 | nIHH | m | AO | no | N218S | *ENST00000344120.4:c.653A>G* | D | P | D | D | L | T | 4/6 |
| 3 | SPRY4 | KS | m | AO | no | R53Q | *ENST00000344120.4:c.158G>A* | T | D | D | D | M | T | 4/6 |
| 4 | SPRY4 | nIHH | m | PPO | no | S241Y | *ENST00000344120.4:c.722C>A* | D | D | D | D | L | T | 4/6 |
|  | GnRHR | nIHH | m | PPO | no | Q106R (Homo) | *ENST00000226413.4:c.317A>G* | D | D | D | A | L | T | 4/6 |
| 5 | SEMA7A | nIHH | m | AO | no | R622H | *ENST00000261918.9:c.1865G>A* | T | B | N | N | N | T | 0/6 |
|  | SEMA3E | nIHH | m | AO | no | N432S | *new* | T | B | D | D | L | T | 2/6 |
| 6 | SEMA3E | nIHH | m | PPO | no | M681V | *ENST00000307792.8:c.2041A>G* | T | B | D | D | M | T | 3/6 |
| 7 | SEMA3E | nIHH | m | AO | yes | A200P | *new* | T | B | D | D | N | T | 2/6 |
| 8 | SEMA7A | nIHH | m | AO | no | N177S | *ENST00000261918.9:c.530A>G* | T | P | N | D | L | T | 2/6 |
| 9 | HESX1 | nIHH | m | AO | no | V129I | *ENST00000295934.7:c.385G>A* | T | P | D | D | N | D | 4/6 |
| 10 | FGFR1 | KS | m | PPO | no | R470C | *ENST00000447712.6:c.1408C>T* | D | B | D | D | L | D | 4/6 |
| 11 | FGFR1 | nIHH | m | AO | no | V248M | *ENST00000447712.6:c.742G>A* | T | D | D | D | L | D | 4/6 |
|  | SEMA3A | nIHH | m | AO | no | T670R(Comp-hetero) | *ENST00000265362.8:c.2009C>G* | T | B | D | D | L | T | 2/6 |
|  | SEMA3A | nIHH | m | AO | no | T664S  (Comp-hetero) | *ENST00000265362.8:c.1991C>G* | T | B | D | D | L | T | 2/6 |
|  | CHD7 | nIHH | m | AO | no | V110I | *ENST00000423902.6:c.328G>A* | T | P | N | D | L | T | 2/6 |
| 12 | SEMA3A | nIHH | m | PPO | no | R637H | *ENST00000265362.8:c.1910G>A* | T | D | D | D | M | T | 4/6 |
| 13 | PROKR2 | nIHH | m | AO | no | V131I | *ENST00000217270.3:c.391G>A* | T | B | N | D | L | T | 1/6 |
| 14 | FLRT3 | KS | m | AO | no | G379R | *ENST00000341420.4:c.1135G>A* | D | P | D | D | L | T | 4/6 |
|  | HS6ST1 | KS | m | AO | no | R382W | *ENST00000259241.6:c.1144C>T* | D | D | N | D | M | D | 5/6 |
| 15 | CHD7 | nIHH | m | AO | no | L2806V | *ENST00000524602.5:c.2269C>G* | T | P | D | D | L | D | 4/6 |
|  | DUSP6 | nIHH | m | AO | no | S182F | *ENST00000279488.7:c.545C>T* | D | D | D | D | L | T | 4/6 |
| 16 | IL17RD | KS | m | PPO | no | M658V | *ENST00000296318.11:c.1972A>G* | D | B | D | D | L | T | 3/6 |
| 17 | SOX10 | nIHH | m | AO | no | A332T | *ENST00000360880.6:c.994G>A* | T | P | D | D | M | D | 5/6 |
| 18 | ANOS1 | KS | m | PPO | yes | H672R | *ENST00000262648.7:c.2015A>G* | T | P | D | N | M | T | 3/6 |
|  | GnRH2 | KS | m | PPO | yes | R49K | *ENST00000245983.6:c.146G>C* | T | B | N | N | L | T | 0/6 |
| 19 | PROKR2 | nIHH | m | AO | yes | R85H | *ENST00000217270.3:c.254G>T* | D | D | D | D | L | T | 4/6 |
| 20 | SPRY4 | nIHH | m | AO | yes | C209Y | *ENST00000344120.4:c.626G>A* | D | B | D | D | M | T | 4/6 |
| 21 | CHD7 | nIHH | m | AO | yes | Ala685_Lys686dup | *ENST00000423902.6:c.2053_2058dup* | . | . | . | . | . | . |  |
| 22 | GnRHR | nIHH | m | PPO | yes | Q106R | *ENST00000226413.4:c.317A>G* | D | D | D | A | L | T | 4/6 |
| 23 | GnRH2 | nIHH | s | PPO | yes | R49K | *ENST00000245983.6:c.146G>C* | T | B | N | N | L | T | 0/6 |
| 24 | HS6ST1 | KS | s | AO | no | E338K | *ENST00000259241.6:c.1012G>C* | T | D | . | D | L | T | 1/5 |
|  | GnRH1 | KS | s | AO | no | C63Y | *ENST00000276414.4:c.188G>A* | D | D | . | D | . | T | 3/4 |
|  | CHD7 | KS | s | AO | no | K729E | *ENST00000423902.6:c.2185A>G* | T | D | D | D | L | T | 3/6 |
| 25 | SEMA3A | KS | s | AO | no | V435I | *ENST00000436949.5:c.1303G>A* | T | B | D | D | M | T | 3/6 |
|  | CHD7 | KS | m | AO | no | A1814V | *new* | D | B | D | D | M | D | 5/6 |
|  | KISS1R | KS | s | AO | no | T155K | *new* | T | D | D | D | M | T | 4/6 |
| 26 | GnRHR | nIHH | s | AO | no | K77E | *ENST00000226413.4:c.229A>G* | D | D | D | N | M | T | 4/6 |
| 27 | FGF8 | KS | m | AO | no | P26L | *ENST00000320185.6:c.77C>T* | D | B | U | A | L | T | 2/6 |
| 28 | CHD7 | nIHH | m | AO | yes | P568T | *ENST00000423902.6:c.1702C>A* | T | P | D | D | L | T | 3/6 |
| 29 | CHD7 | nIHH | m | AO | no | V1988A | *new* | T | B | N | N | N | D | 1/6 |
|  | FLRT3 | nIHH | m | AO | no | P598S | *ENST00000341420.4:c.1792C>T* | T | B | D | D | L | T | 2/6 |
| 30 | FGFR1 | KS | s | PPO | yes | S353C | *new* | D | D | D | D | M | T | 5/6 |
| 31 | FGFR1 | nIHH | s | PPO | yes | R744T | *new* | D | D | D | D | H | D | 6/6 |
| 32 | PROK2 | nIHH | s | PPO | yes | T31I | *ENST00000295619.3:c.92C>T* | D | D | D | D | M | D | 6/6 |
| 33 | ANOS1 | KS | s | PPO | yes | R272X | *new* | . | . | . | . | . | . |  |
| 34 | SEMA3A | nIHH | s | PPO | yes | V435I | *ENST00000265362.8:c.1303G>A* | T | B | D | D | M | T | 3/6 |
| 35 | SEMA7A | nIHH | s | PPO | yes | IVS 3+7 bp G>C | *.* | . | . | . | . | . | . |  |

IHH: Isolated hypogonadotropic hypogonadism; nIHH: normosmic IHH; KS: Kallmann syndrome; Testosterone deficit: m = mild (>3.5 nMol/L), s = severe (≤3.5 nMol/L); AO: Adult Onset IHH; PPO: Pre-pubertal onset IHH; if a variant was present in homozygosity it was reported as “Homo”. MT: Mutation Taster; MA: Mutation Assessor. Variants with a demonstrated functional impact or predicted to be deleterious in  ≥4/6 in silico programmes were highlighted in grey. We considered to be deleterious also variants that result in a truncated protein whereas we excluded intronic variants from this evaluation. D: Deleterious; P: Possibly damaging; A: “Disease_causing_auomatic”; M: Medium impact; T: Tolerated; B: Begnin; L: low impact; N: Neutral; U: Unknown, as reported at: https://annovar.readthedocs.io/en/latest/user-guide/filter/.
