# Supplementary material for: Evidence for a Common Genetic Origin of Classic and Milder Adult-Onset Forms of Isolated Hypogonadotropic Hypogonadism
Source: J Clin Med. 2019 Jan 21;8(1):126. doi: 10.3390/jcm8010126 (PMC6352096; doi:10.3390/jcm8010126)
Supplement: Supplementary file 1 [file jcm-08-00126-s001.zip › Supplementary Table S2.docx]

**Table S2.** Gene allelic variant list in classic IHH patient group.

| **ID** | **Gene** | **Olfactory Defect** | **Variant** | **SIFT** | **Polyphen2** | **LRT** | **MT** | **MA** | **FATHMM** | **Score** |
| --- | --- | --- | --- | --- | --- | --- | --- | --- | --- | --- |
| 1 | PROKR2 | KS | L173R | D | D | D | A | L | T | 4/6 |
|  | FEZF1 | KS | K128Q | D | D | D | D | L | T | 4/6 |
| 2 | ANOS1 | KS | V587L | T | D | D | D | L | T | 3/6 |
| 3 | ANOS1 | KS | P293AfsX17 | . | . | . | . | . | . |  |
| 4 | FGFR1 | nIHH | R822C | D | D | D | D | L | T | 4/6 |
| 5 | HESX1 | nIHH | V74M | T | B | N | N | N | D | 1/6 |
| 6 | ANOS1 | KS | V182AfsX3 | . | . | . | . | . | . |  |
| 7 | FGF8 | KS | G33V | T | B | U | D | L | D | 2/6 |
|  | SEMA3A | KS | N153S | T | B | D | D | L | T | 2/6 |
| 8 | FGFR1 | KS | M456I | T | B | D | D | M | D | 4/6 |
|  | TACR3 | KS | R425Q | T | B | N | N | M | T | 1/6 |
| 9 | GnRHR | nIHH | Q106R | D | D | D | A | L | T | 4/6 |
| 10 | FGF8 | nIHH | R195W | D | D | D | D | M | D | 6/6 |
| 11 | KISS1R | KS | M136R | D | D | D | D | H | T | 5/6 |
|  | CHD7 | KS | F1425L | D | P | D | D | L | T | 4/6 |
| 12 | GnRH1-2 | nIHH | L18R | D | D | N | N |  | T | 2/5 |
| 13 | KAL-1 | KS | R191X | . | . | . | . | . | . |  |
| 14 | PROKR2 | nIHH | R85C | D | D | D | D | H | T | 5/6 |
| 15 | SEMA3A | KS | V435I | D | B | D | D | M | T | 4/6 |
| 16 | FGFR1 | KS | R250Q | D | D | D | A | M | T | 5/6 |
| 17 | IL17RD | nIHH | T649M | D | P | N | N | N | T | 2/6 |
| 18 | SOX10 | nIHH | P347T | T | B | N | N | L | D | 1/6 |
| 19 | IL17RD | nIHH | A642P | D | P | N | N | L | T | 2/6 |
| 20 | PROKR2 | KS | R85C Homo | D | D | D | D | H | T | 5/6 |
| 21 | SOX10 | KS | E3X | . | . | . | . | . | . |  |
| 22 | FGFR1 | nIHH | K195R | T | B | D | D | L | T | 2/6 |
| 23 | ANOS1 | KS | IVS13+2bpT>A (splicing) | . | . | . | . | . | . |  |
| 24 | PROK2 | KS | S49I | D | D | D | D | M | D | 6/6 |
| 25 | PROKR2 | KS | L173R | D | D | D | A | L | T | 4/6 |
|  | GnRHR | KS | Q106R | T | D | D | A | L | T | 3/6 |
| 26 | FGFR1 | KS | G97V | D | D | D | D | M | T | 5/6 |
| 27 | PROKR2 | KS | R382K | T | B | D | D | L | T | 2/6 |
|  | CHD7 | KS | N2891S | T | B | D | D | N | T | 2/6 |
| 28 | KISS1R | nIHH | C389X |  |  | N | N |  |  | 0/2 |
| 29 | HS6ST1 | KS | Y319N | . | D | D | D | L | T | 3/5 |
| 30 | GnRHR | nIHH | G99E Homo | D | D | D | D | L | T | 4/6 |
| 31 | FLRT3 | nIHH | Q378H | T | B | D | D | L | T | 2/6 |
| 32 | ANOS1 | KS | W76GfsX21 | . | . | . | . | . | . |  |
|  | SEMA7A | KS | V488L | T | B | N | D | L | T | 1/6 |
| 33 | PROKR2 | KS | R268C | D | D | D | D | M | T | 5/6 |
|  | NELF (NSMF) | KS | Q496L | D | D | D | D | L | T | 4/6 |
| 34 | CHD7 | nIHH | R758C | D | D | D | D | L | T | 4/6 |
| 35 | PROKR2 | KS | R135C | D | D | D | D | L | T | 4/6 |
|  | SEMA3A | KS | V435I | D | B | D | D | M | T | 4/6 |
|  | SPRY4 | KS | C209Y | D | B | D | D | M | T | 4/6 |
| 36 | WDR11 | KS | M769V | T | B | D | D | N | D | 3/6 |
| 37 | GnRHR | nIHH | N10K | T | B | N | N | N | T | 0/6 |
|  | GnRHR | nIHH | Q11K | T | B | N | N | N | T | 0/6 |
|  | GnRHR | nIHH | Q106R | T | D | D | A | L | T | 3/6 |
| 38 | FGFR1 | nIHH | H717TfsX15 | . | . | . | . | . | . |  |
| 39 | ANOS1 | KS | H459RfsX5 | . | . | . | . | . | . |  |
| 40 | NELF (NSMF) | nIHH | R281H | D | D | D | D | M | T | 5/6 |
| 41 | GnRHR | nIHH | A86P | D | D | D | D | M | T | 5/6 |
|  | GnRHR | nIHH | A86V | D | D | D | D | M | T | 5/6 |
| 42 | PROKR2 | KS | R268H | D | D | D | D | M | T | 5/6 |
| 43 | SEMA3A | nIHH | R637H | D | D | D | D | M | T | 5/6 |
| 44 | SEMA3A | KS | R66W | D | D | D | D | M | T | 5/6 |
| 45 | FGFR1 | nIHH | C381LfsX27 | . | . | . | . | . | . |  |
| 46 | PROK2 | nIHH | M1R | D | D | N | D |  | D | 4/5 |
| 47 | PROKR2 | nIHH | V158I | T | D | D | D | L | T | 3/6 |
|  | CHD7 | nIHH | G1338S | D | D | D | D | H | D | 6/6 |
| 48 | CHD7 | nIHH | P277L | D | D | D | D | L | D | 5/6 |
| 49 | FGFR1 | nIHH | Q553X | . | . | . | . | . | . |  |
| 50 | KISS1R | nIHH | K116E Homo | D | D | U | D | M | T | 4/6 |
| 51 | HS6ST1 | nIHH | P218S | T | D | D | D | L | T | 3/6 |
| 52 | PROKR2 | KS | L173R | D | D | D | A | L | T | 4/6 |
|  | TAC3R | KS | A449T | T | B | N | N | N | T | 0/6 |
| 53 | CHD7 | KS | IVS6+5 | . | . | . | . | . | . | . |

IHH: Isolated hypogonadotropic hypogonadism; nIHH: normosmic IHH; KS: Kallmann syndrome; MT: Mutation Taster; MA: Mutation Assessor. Variants with a demonstrated functional impact or predicted to be deleterious in  ≥4/6 in silico programmes were highlighted in grey. We considered to be deleterious also variants that result in a truncated protein whereas we excluded intronic variants from this evaluation. D: Deleterious; P: Possibly damaging; A: “Disease_causing_auomatic”; M: Medium impact; T: Tolerated; B: Begnin; L: low impact; N: Neutral; U: Unknown, as reported at: https://annovar.readthedocs.io/en/latest/user-guide/filter/.
