# Supplementary material for: Evidence for a Common Genetic Origin of Classic and Milder Adult-Onset Forms of Isolated Hypogonadotropic Hypogonadism
Source: J Clin Med. 2019 Jan 21;8(1):126. doi: 10.3390/jcm8010126 (PMC6352096; doi:10.3390/jcm8010126)
Supplement: Supplementary file 1 [file jcm-08-00126-s001.zip › Supplementary Table S3.docx]

**Table S3.** Gene allelic variant list in controls.

| **ID** | **Gene** | **Variant** | **SIFT** | **Polyphen2** | **LRT** | **MT** | **MA** | **FATHMM** | **SCORE** |
| --- | --- | --- | --- | --- | --- | --- | --- | --- | --- |
| 1 | SPRY4 | p.V16I | T | . | N | N | . | T | 0/4 |
| 2 | SEMA3E | p.S701I | T | B | N | N | N | T | 0/6 |
| 3 | GnRHR | p.Q106R | T | D | D | A | L | T | 3/6 |
| 4 | GnRH2 | R49K | T | B | N | N | L | T | 0/6 |
|  | ANOS1 | p.S511Y | T | B | N | N | L | T | 0/6 |
| 5 | FEZF1 | p.S85G | T | B | N | N | N | T | 0/6 |
| 6 | KISS1R | p.P16L | T | B | N | N | N | T | 0/6 |
| 7 | ANOS1 | p.H672R | T | P | D | N | L | T | 2/6 |
| 8 | CHD7 | p.R312L | T | P | N | D | L | T | 2/6 |
| 9 | CHD7 | p.R2719K | D | P | D | D | M | T | 5/6 |
| 10 | HS6ST1 | p.R306Q | . | D | N | D | L | D | 3/5 |
| 11 | IL17RD | p.T684M | T | B | N | N | L | T | 0/6 |
|  | TACR3 | p.A449S | T | B | D | N | N | T | 1/6 |
| 12 | SEMA3A | p.G452R | D | D | D | D | M | T | 5/6 |
| 13 | SEMA3E | p.D303H | D | D | D | D | L | T | 4/6 |
| 14 | WDR11 | p.R266G | T | D | D | D | M | D | 5/6 |

IHH: Isolated hypogonadotropic hypogonadism; nIHH: normosmic IHH; KS: Kallmann syndrome; MT: Mutation Taster; MA: Mutation Assessor. Variants with a demonstrated functional impact or predicted to be deleterious in  ≥4/6 in silico programmes were highlighted in grey. We considered to be deleterious also variants that result in a truncated protein whereas we excluded intronic variants from this evaluation. D: Deleterious; P: Possibly damaging; A: “Disease_causing_auomatic”; M: Medium impact; T: Tolerated; B: Begnin; L: low impact; N: Neutral; U: Unknown, as reported at: <https://annovar.readthedocs.io/en/latest/user-guide/filter/>.
